# An exploratory study of imaging diagnostic clues for overhanging facial nerve in ultra-high-resolution CT

**DOI:** 10.1007/s00405-023-07879-0

**Published:** 2023-02-27

**Authors:** Zhengyu Zhang, Ruowei Tang, Qian Wu, Pengfei Zhao, Zhenghan Yang, Zhenchang Wang

**Affiliations:** 1 Department of Radiology, Beijing Friendship Hospital, Capital Medical University, 95 Yong An Road, Western District, Beijing, 100050 China; 2 Department of Otolaryngology, Head and Neck Surgery, Beijing Friendship Hospital, Capital Medical University, Beijing, China

**Keywords:** Facial nerve, Oval window, Ear, Diagnostic imaging, Tomography, X-ray Computed

## Abstract

**Purpose:**

Overhanging facial nerve (FN) may be challenging in imaging diagnosis. The purpose of the study is to investigate the imaging clues for overhanging FN near the oval window on ultra-high-resolution computed tomography (U-HRCT) images.

**Methods:**

Between October 2020 and August 2021, images of 325 ears (276 patients) were included in the analysis obtained by an experimental U-HRCT scanner. On standard reformatted images, the morphology of FN was evaluated and its position was quantitatively measured using the following indices: protrusion ratio (PR), protruding angle (A), position of FN (P-FN), distance between FN and stapes (D-S), and distance between FN and anterior and posterior crura of stapes (D-AC and D-PC). According to the FN morphology in imaging, images were divided into overhanging FN group and non-overhanging FN group. Binary univariate logistic regression analysis was used to identify the imaging indices independently associated with overhanging FN.

**Results:**

Overhanging FN was found in 66 ears (20.3%), which manifested as downwards protrusion of either local segment (61 ears, 61/66) or the entire course near the oval window (5 ears, 5/66). D-AC [odds ratio: 0.063, 95% CI 0.012–0.334, *P* = 0.001) and D-PC (odds ratio: 0.008, 95% CI 0.001–0.050, *P* = 0.000) were identified as independent predictors of FN overhang (area under the curve: 0.828 and 0.865, respectively).

**Conclusion:**

Abnormal morphology of the lower margin of FN, D-AC and D-PC on U-HRCT images provide valuable diagnostic clues for FN overhang.

## Introduction

The tympanic segment of the facial nerve (FN) is one of the important structures in the medial wall of the tympanic cavity in the temporal bone. It is located at the upper edge of the oval window niche, above the stapes. However, the position of the FN may vary, which is one of the key challenges hindering adequate exposure in oval window-related surgery [1]. The low position of the FN, also referred to as overhanging FN, may cause narrowing of the oval window niche, making surgical treatment challenging [2]. Iatrogenic facial paralysis is a very rare but always threatening complication of oval window-related surgery [1, 3–5]. Preoperative imaging can be used to provide information regarding FN anatomy. However, the diagnosis of overhanging FN is challenging for non-otological radiologists, and heavily depends on the expertise of the viewer. A good anatomical knowledge of FN and objective measurement methods can provide in-depth characterization of overhanging FN and improve the diagnostic accuracy.

Computed tomography (CT) is widely used in temporal bone imaging. However, to the best of our knowledge, there is a paucity of studies that have focused on overhanging FN near the oval window [6, 7]. We did not find any detailed imaging studies of overhanging FN. This may be attributable to the limited spatial resolution of CT for delineating the fine anatomical changes of the FN. Ultra-high-resolution computed tomography (U-HRCT), a newly designed imaging device based on cone-beam computed tomography (CBCT), features a significantly higher spatial resolution [8]. It has been shown to provide better delineation of stapes and the FN than conventional 128-section multislice CT (MSCT) [9].

The purpose of this study was to identify the imaging diagnostic clues for overhanging FN using this new U-HRCT.

## Methods

### Study population

This retrospective, single-center study was approved by the medical ethics committee of the hospital (IRB: 2020-P2-061-02) and informed consent was obtained from all the participants. Between October 2020 and August 2021, U-HRCT images of ears of patients were collected and those who qualified the following inclusion and exclusion criteria were enrolled in the study. The inclusion criteria were: (1) age ≥ 18 years; (2) absence of any symptoms of facial nerve lesion; (3) without lesions in the tympanic cavity and inner ear in images. The exclusion criterion was poor image quality (presence of motion artifacts or incomplete images).

### Image acquisition

The ears were examined by an experimental U-HRCT scanner (Ultra3D-SE, LargeV, Beijing, China). The device was integrated with a small-focus X-ray generator, a high-stability motion control unit, and a high-resolution flat-panel detector (unit size: 0.0748 mm × 0.0748 mm). Unilateral temporal bone images were obtained on each scan. The scan protocols were as follows: 100 kV; 3.5 mA; voxel size: 0.1 mm × 0.1 mm × 0.1 mm; field of view: 65 mm × 65 mm. exposure time: 40 s; the number of images: 370.

All images were exported in DICOM file format and archived in a three-dimensional workstation using special software (RadiAnt DICOM Viewer, RadiAnt, Poland) for image analysis.

### Image analysis

(1) Quantitative measurement of FN position

Using the RadiAnt software, standard coronal images of tympanic segment of the FN were reformatted. The coronal plane was perpendicular to the lateral semicircular canal, and this is a commonly used reformatted section in clinical work [10–12].

**Fig. 1:**
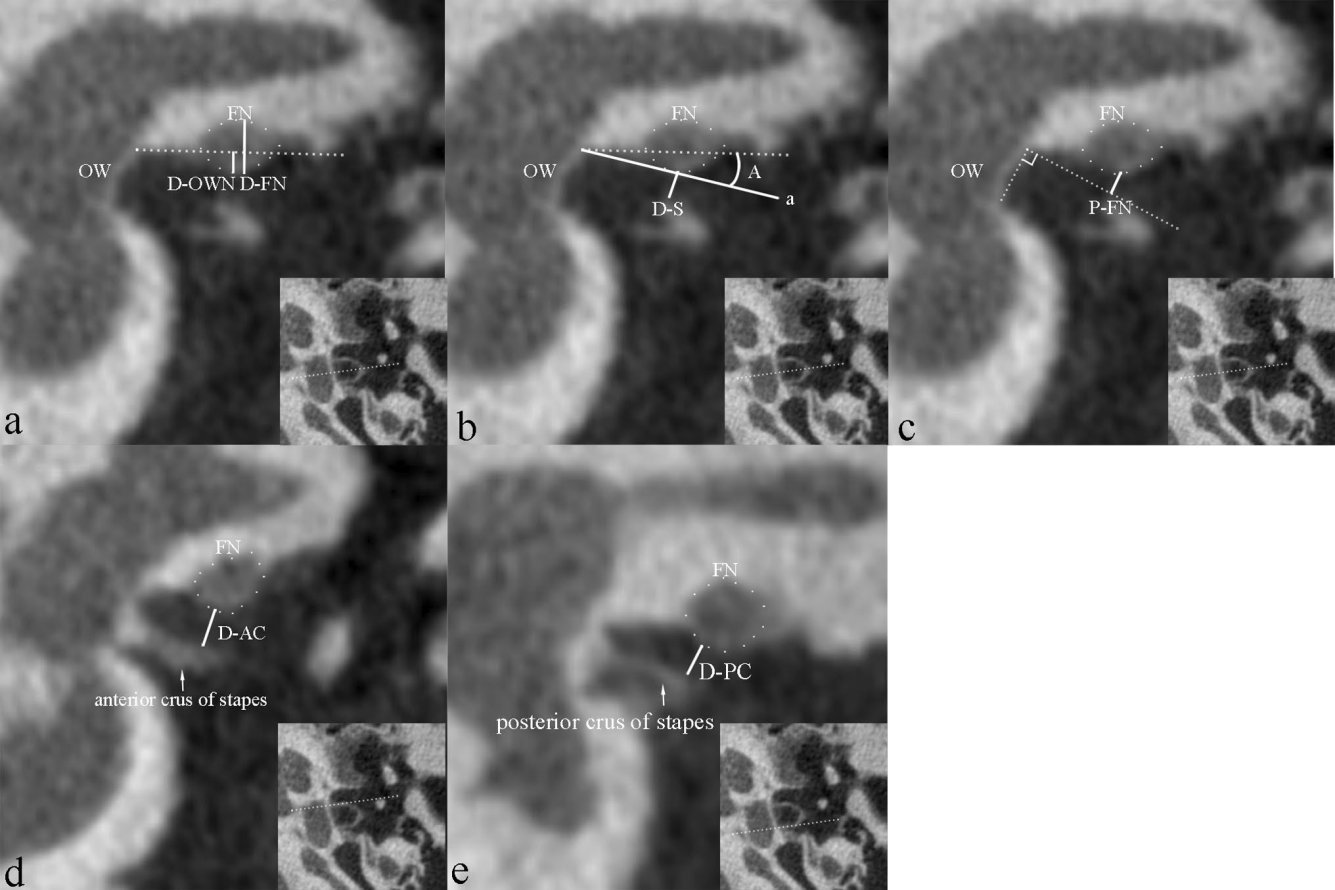
Position measurement of the facial nerve (FN) near oval window on U-HRCT standard coronal images. **a** The image passing through the middle of the stapes footplate; D-FN and D-OWN are measured. The dotted line is the upper border of oval window niche. **b** The same section as a, A is measured. Line a is the line connecting the lower margin of FN and the upper edge of oval window; dotted line is the upper border of oval window niche. D-S is also measured. **c** The same section as a, P-FN is measured. **d** The image passing through the anterior crus of stapes, D-AC is measured. **e** The image passing through the posterior crus of stapes, D-PC is measured. *OW* oval window, *FN* facial nerve

To evaluate the position of the tympanic segment of the FN, the following indices were measured on coronal images (Fig. 1):

(a) Protrusion ratio (PR). The vertical diameter of FN (D-FN) and protrusion distance relative to upper border of oval window niche (D-OWN) were measured; PR was calculated as D-OWN/D-FN. The measurements were done on the image passing through the middle of the stapes footplate.

(b) Protrusion angle (A). The angle between the line connecting the lower margin of FN and the upper edge of oval window and the line of the upper border of oval window niche was measured on the same image as point (a).

(c) Position of FN (P-FN). The position of FN relative to the oval window was measured. The distance between the lower margin of FN and the plane of the upper point of oval window was measured on the same image as point (a).

(d) Distance between FN and stapes (D-S). The distance between the lower point of FN and the stapes was measured on the same image as point (a).

(e) Distance between FN and anterior and posterior crura of stapes (D-AC and D-PC), which was measured on the images demonstrating the anterior or posterior crus.

(2) Overhanging FN observation

**Fig. 2:**
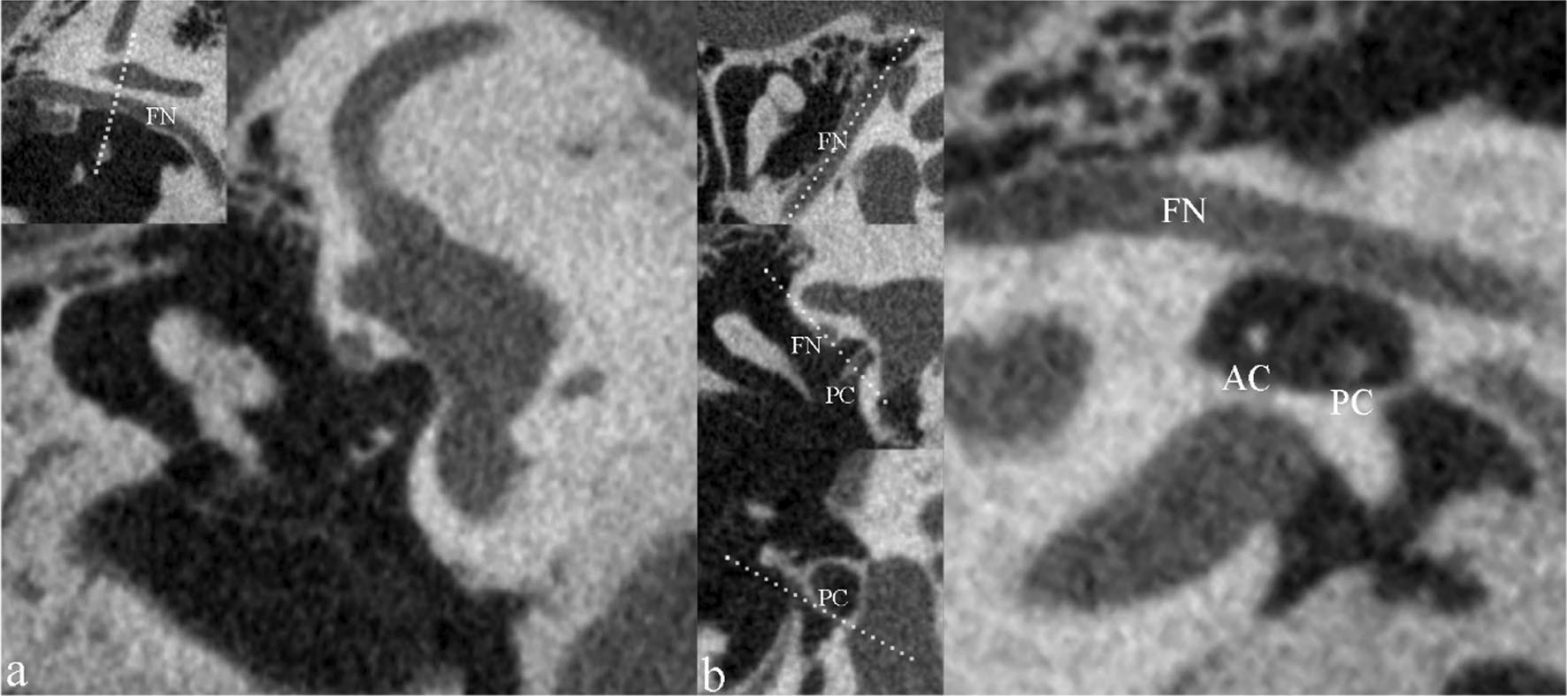
Reformation of the sections of tympanic segment of facial nerve (FN). The coronal plane (**a**) is perpendicular to the tympanic segment of FN. The oblique sagittal plane (**b**) is perpendicular to the posterior crus of the stapes on the basis of parallel to the tympanic segment of FN. *AC* anterior crus of stapes, *PC* posterior crus of stapes

Using the RadiAnt software, coronal and oblique sagittal images were reformatted and used to observe the tympanic segment of FN. The coronal plane for FN was perpendicular to the tympanic segment of FN. The oblique sagittal plane was parallel to the tympanic segment of the FN and was perpendicular to the posterior crus of the stapes (Fig. 2). The assessment range of the tympanic segment of FN near the oval window was defined as the region between the cochleariform process and the anterior edge of the pyramidal eminence.

Based on the morphological description of overhanging FN reported in published literature [5, 13], when the lower margin of the FN near the oval window was partially or completely moving downward on images, it was defined as overhanging FN. The observation was made on the oblique sagittal and coronal images reformatted for FN.

According to the observation results, the FN images were divided into two groups: overhanging FN group and non-overhanging FN group. Then, the two groups were statistically analyzed.

The U-HRCT images were independently evaluated by two radiologists with 5-year experience (R.W.T.) and 20-year experience (Z.Y.Z.), respectively, in head and neck imaging diagnosis. Continuous variables presented as the average of the results measured by the two radiologists. If there was a difference in the observation of prolapse, the conclusion was drawn by consensus.

### Statistical analysis

Statistical analysis was conducted using IBM SPSS Statistics version 22.0. The Kolmogorov–Smirnov test was used to test for normality of distribution of variables. Continuous variables are expressed as mean ± standard deviation or median (interquartile range) depending on the normality of distribution. Interrater repeatability was assessed by calculating the intraclass correlation coefficient (ICC). The ICC values were interpreted using the benchmarks suggested by Landis and Koch [14]: 0.81–1 almost perfect, 0.61–0.8 substantial, 0.41–0.6 moderate, 0.21–0.4 fair, and 0–0.2 slight.

Between-group differences with respect to continuous variables were assessed using the Mann–Whitney *U* test, while those with respect to categorical variables were assessed using the Chi-squared test. Variables that showed significant between-group differences (*P* < 0.05) were selected for multivariate analysis. Binary univariate logistic regression analysis according to a forward stepwise method was used to identify the imaging indices independently associated with overhanging FN. MedCalc (version 19.0, Ostend, Belgium) was used to plot the receiver operator characteristic (ROC) curve and the area under the curve (AUC) was calculated to analyze the diagnostic performance of different imaging indices for overhanging FN. *P* values < 0.05 were considered indicative of statistical significance.

## Results

U-HRCT images of 340 ears (285 participants) were initially evaluated. Motion artifacts were found in the images of 15 ears (9 participants) which qualified the exclusion criteria. Finally, 325 ears (left 135, right 190) in 276 participants (128 male, 148 female; mean age 40.4, age range 18–83) were enrolled in this study.

### Imaging morphology of overhanging FN

**Fig. 3:**
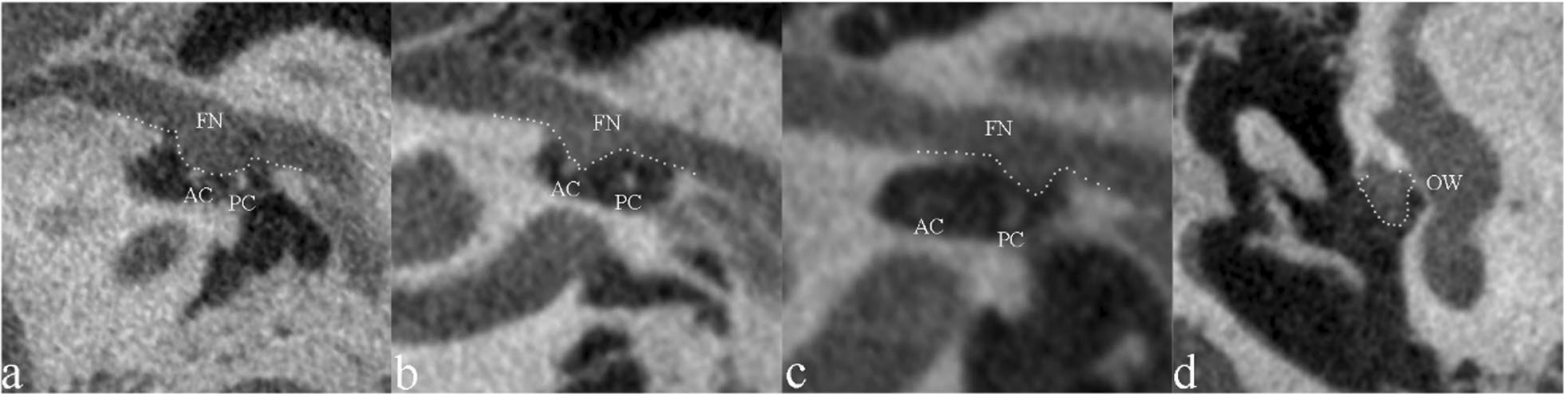
Imaging morphology of overhanging facial nerve (FN) on U-HRCT images. **a** Oblique sagittal image of a 20-year-old woman showing the entire course of the right FN near the oval window protruding downwards (dotted line), touching the anterior crus (AC) and posterior crus (PC) of stapes. **b** Oblique sagittal image of a 35-year-old woman showing the anterior portion of the right FN near the oval window protruding downwards (dotted line), touching the AC of stapes. **c** Oblique sagittal image of a 40-year-old man showing the posterior portion of FN near the oval window protruding downwards (dotted line), close to the PC of stapes. **d** Coronal image of the case shown in image B showing irregular morphology and downward protrusion of FN, blocking the oval window (OW)

The morphology of the tympanic segment of FN near the oval window was well demonstrated on oblique sagittal images. In 66 ears (66/325, 20.3%), the lower margin of the FN protruded downwards partially (61 ears, 61/66) or totally (5 ears, 5/66) (Fig. 3), which is the direct sign of overhanging FN. On the coronal images reformatted for FN, all the overhanging FN exhibited a sudden change in morphology of FN, beyond the original position (Fig. 3). These 66 ears were classified in overhanging FN group. The other 259 ears were included in non-overhanging FN group. The specific site of protrusion of FN and the demographic characteristics of the two groups are summarized in Table 1.

**Table 1:** Site of overhanging FN and demographic features in the two groups (*n* = 325 ears)

	Overhanging FN group (*n* = 66)	Non-overhanging FN group (*n* = 259)	*P* value
Site			
Total	5	None	NA
Local			
Anterior	5	None	NA
Middle	15	None	NA
Posterior	41	None	NA
Side			
Left	29	106	0.657
Right	37	153	
Age (years)	38.3 ± 15.8	39.2 ± 14.9	0.567
Sex			
Male	29	125	0.530
Female	37	134	

*FN* facial nerve

### Quantitative indices of overhanging FN

**Fig. 4:**
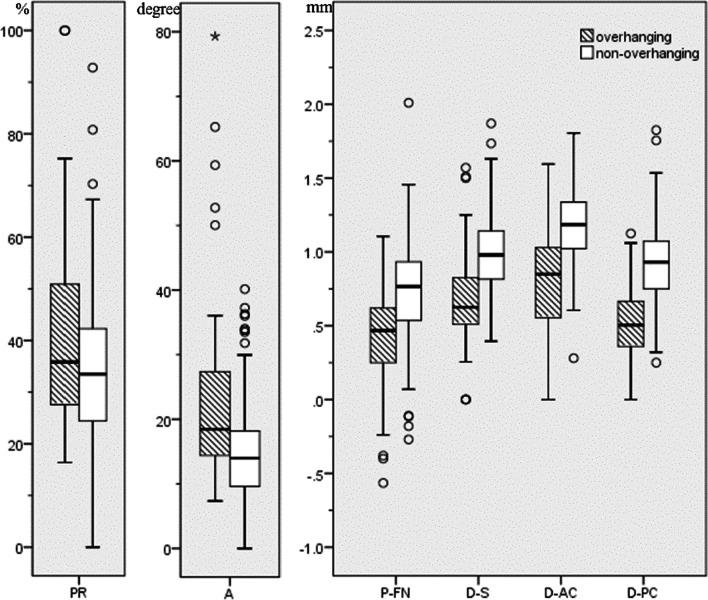
Boxplots showing the measured indices in the overhanging facial nerve (FN) and non-overhanging FN groups

The values of the indices are listed in Table 2. The protrusion ratio (PR) and the angle (A) of FN protrusion relative to upper edge of oval window niche were significantly larger in the overhanging FN group (*P* < 0.05). The distances between the lower margin of the FN and the stapes, anterior and posterior crura were significantly smaller in the overhanging FN group (*P* = 0.000) (Table 2) (Fig. 4).

**Table 2:** Measured indices in the overhanging and non-overhanging FN groups

Indices	ICC	Overhanging FN		Non-overhanging FN		M–W *U* test *P* value
		Value	K–S test *P* value	Value	K–S test *P* value	
PR (%)	0.752	35.9 (27.5, 51.2)	0.001	33.5 (24.3, 42.3)	0.015	0.023
A (degree)	0.781	18.5 (14.4, 27.4)	0.000	14.0 (9.6, 18.3)	0.013	0.000
P-FN (mm)	0.706	0.4 ± 0.3	0.2	0.8 (0.5, 0.9)	0.024	0.000
D-S (mm)	0.724	0.7 ± 0.3	0.072	1.0 ± 0.3	0.2	0.000
D-AC (mm)	0.863	0.9 (0.5, 1.0)	0.001	1.2 ± 0.2	0.2	0.000
D-PC (mm)	0.835	0.5 ± 0.3	0.2	0.9 ± 0.2	0.2	0.000

*PR* protrusion ratio of facial nerve, *A* facial nerve protruded angle relative to the upper edge of oval window niche, *P-FN* positon of facial nerve relative to the oval window, *D-S* distance between facial nerve and stapes, *D-AC* distance between facial nerve and the anterior crus of stapes, *D-PC* distance between facial nerve and the posterior crus of stapes

**Fig. 5:**
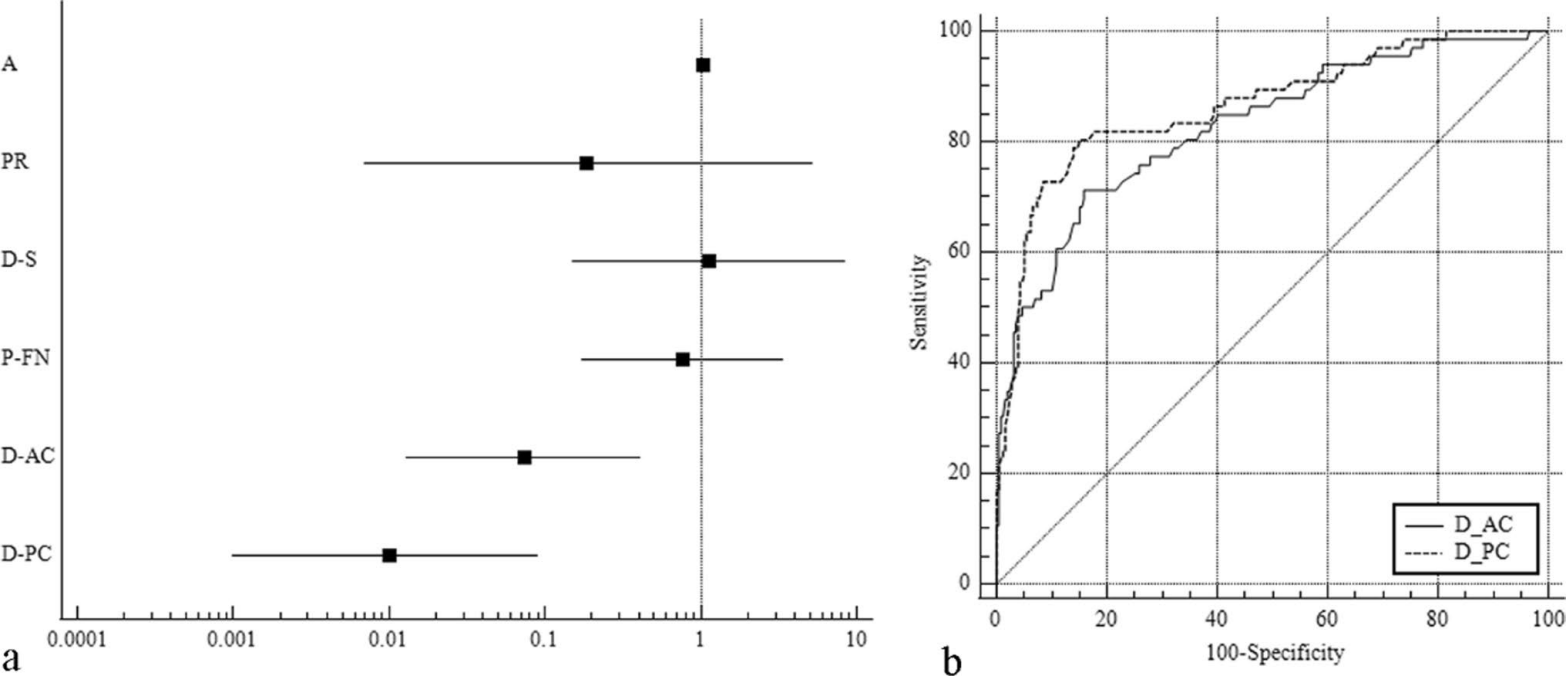
**a** Forest plot of binary logistic regression results of the measured indices;** b** receiver operator characteristic (ROC) curves of D-AC and D-PC for the diagnosis of overhanging facial nerve

All the measured indices were significantly different in the overhanging FN group and were included in the binary univariate logistic regression model. Finally, D-AC [odds ratio (OR): 0.063, 95% CI 0.012–0.334, *P* = 0.001) and D-PC (OR: 0.008, 95% CI 0.001–0.050, *P* = 0.000) were identified as independent predictors of FN overhang. On ROC curve analysis, the AUCs of D-AC and D-PC were 0.828, and 0.865, respectively (*P* < 0.001) (Fig. 5). The sensitivity and specificity of D-AC and D-PC for predicting FN overhang, and the optimal cutoff values are shown in Table 3.

**Table 3:** Predictive efficiency of D-AC and D-PC for overhanging FN

	Sensitivity (%)	Specificity (%)	Cutoff value
D-AC	71.2	84.2	≤ 0.975 mm
D-PC	80.3	84.9	≤ 0.69 mm

*DAC* distance between facial nerve and the anterior crus of stapes, *D–PC* distance between facial nerve and the posterior crus of stapes

## Discussion

In the present study, we used a new device U-HRCT to demonstrate the imaging characteristics of overhanging FN in the oval window niche region. Using the direct imaging sign of overhanging FN as the standard, we identified the quantitative indices with high predictive value for FN overhang. Our findings may facilitate preoperative diagnosis of overhanging FN, which is important for oval window-related surgery.

FN overhang refers to a kind of variation in the course of the FN, which appears as variable degrees of inferior displacement, in close proximity to the stapes. In cases with FN overhang, the FN may partially or completely cover the stapes [5, 13, 15]. The overhanging FN may narrow the oval window increasing the difficulty in performing surgery. Therefore, FN overhang is of much concern for otology surgeons and the associated challenges are described in surgical studies [1, 4, 5, 13, 16]. Preoperative detection of the change in the anatomical course of the FN may help the surgeon in predicting an intraoperative challenge. Temporal bone imaging is the best method to evaluate the FN canal [7, 10, 17, 18]. However, the imaging characteristics of overhanging FN are not well characterized in the contemporary literature. In the current study, we found that the U-HRCT images could demonstrate the downward protrusion of the lower margin of the tympanic segment of FN, which was consistent with the appearance described in previous surgical studies [5, 13]. The degree of protrusion of FN was variable, from slight to severe prolapse enough to cover the stapes, which was in accordance with the previous reports [5, 13]. The reported prevalence of overhanging FN in previous studies ranged from 2.5 to 32%, which were all based on intraoperative observation [2, 13, 19]. In the present study, overhanging FN was identified in 66 ears (66/325, 20.3%) using direct imaging signs. The differences in the prevalence rates may be attributable to differences in research methods and study populations. In our experience, the reconstructed oblique sagittal plane which is parallel to the tympanic segment of the FN and is perpendicular to the posterior crus of the stapes is recommended for better delineation of the overhanging FN. This plane can help detect the abnormal morphology of the lower margin of the FN and can simultaneously display the relationship between the overhanging FN and the stapes. On the coronal images reformatted for FN, overhanging FN should be continuously observed from the front section to the back, and presents as a sudden downward protrusion of FN from its previous course. But these two special reformation methods may be difficult and complicated. While on the commonly used coronal images which are perpendicular to the lateral semicircular canal, the FN is in an oblique section, and some small morphological changes may be difficult to observe. Therefore, we tried to develop quantitative indices to facilitate diagnosis. Our findings suggest that the distance between the FN and the anterior or the posterior crus of the stapes are both good predictors of overhanging FN. If the distance is smaller than 0.975 mm or 0.69 mm, respectively, an overhanging FN should be taken into consideration.

The overhanging FN moves downwards in close proximity to or covering the stapes [1, 4]. In the current study, we measured the FN protrusion ratio, angle, the position of FN relative to the oval window and the distance between FN and stapes to evaluate FN position. These quantitative indices were chosen because they are simple and convenient to measure using precise and reproducible anatomic landmarks, making the evaluation visual and repeatable. The results showed that in ears with overhanging FN, the FN protruded downwards to the oval window niche, and the distances between FN and crura of stapes were significantly smaller, which was consistent with the intraoperative findings reported in previous studies. Among the six indices used in the study, the other four items showed no statistical significance for diagnosis of FN prolapse, although their values differed between the prolapse and non-prolapse groups. To the best of our knowledge, overhanging FN has not been evaluated in images in previous studies. Some scholars have measured the height of oval windows and studied its correlation with the degree of surgical difficulty [4, 10, 20]. Overhanging FN was also described to be one of the main causes leading to oval window niche narrowing; however, there were no overhanging FN measurements. The indices evaluated in this study will facilitate understanding of the imaging manifestations of overhanging FN and provide a basis for diagnosis. In previous studies, overhanging FN was categorized as dehiscent overhanging FN and bony overhanging FN [2, 5, 19]. In the former, the FN canal was dehiscent leading to FN herniation, while in the latter, the FN canal was intact with a low location. Owing to the thin wall of the FN canal, it is typically poorly displayed in images. The results of previous imaging studies on the bone continuity of FN canal have not been consistent [7, 17, 21]. In our study, the FN canal wall was not evaluated to avoid observation errors. In cases with obvious overhanging FN in the present study, the lower margin of the FN was significantly protruded, deviating from the running area of the original FN canal, and the local wall was not demonstrated. We conjectured that this was a manifestation of dehiscent FN canal and overhanging FN. A comparative study with intraoperative findings will be further explored.

Although CT is the preferred method for temporal bone imaging, fine and complex structures are not displayed well because of its limited spatial resolution [6, 22]. U-HRCT is a newly developed high-resolution equipment for temporal bone imaging, which is based on CBCT. CBCT offers advantages over the current-generation 64- or 128-slice MSCT in temporal bone imaging in terms of higher spatial resolution and lower radiation dose [23, 24]. U-HRCT is currently being evaluated in clinical trials. Its spatial resolution is equal to or greater than 4 line pairs per millimeter (l p/mm), with the routine reconstructed voxel size of 0.1 mm × 0.1 mm × 0.1 mm [8]. This ultrahigh spatial resolution helps identify small imaging changes in the temporal bone. In this study, U-HRCT was used to gain clear images of the tympanic segment of FN that allowed us to observe the morphological changes of overhanging FN, which was an imaging supplement to understand overhanging FN.

Some limitations of our study should be acknowledged. First, the overhanging FN was observed in images without comparison with intraoperative findings. The U-HRCT is still an experimental equipment. There were not enough cases for which our findings could be compared with intraoperative findings. The morphology of the overhanging FN described in the study was consistent with our previous experience and literature reports. We will accumulate more imaging-surgical comparison cases for further study. Second, the radiation dose of U-HRCT was not provided in this study. The U-HRCT device used in the study does not have the capability for real-time detection of radiation doses. In a previous study, the radiation dose of this U-HRCT with the same scanning conditions as used in the study was found to be approximately 1/3 of that of conventional MSCT [25]. Lastly, owing to the very small size of the measured anatomical structures, the possibility of measurement error cannot be ruled out. However, to minimize the error, two radiologists separately measured the indices using standardized measurement methods. The ICC values showed moderate to almost perfect agreement.

## Conclusion

We identified some imaging diagnostic clues for overhanging FN in U-HRCT images, which included the abnormal morphology of the lower margin of FN and the distance between FN and the anterior and posterior crura of stapes. All these indices can provide valuable information for imaging diagnosis of overhanging FN.

## Data Availability

No additional data are available.
